# Meta-analysis of the effects of ambient temperature and relative humidity on the risk of mumps

**DOI:** 10.1038/s41598-022-10138-7

**Published:** 2022-04-19

**Authors:** Taiwu Wang, Junjun Wang, Jixian Rao, Yifang Han, Zhenghan Luo, Lingru Jia, Leru Chen, Chunhui Wang, Yao Zhang, Jinhai Zhang

**Affiliations:** 1 Department of Infectious Disease Prevention and Control, Center for Disease Control and Prevention of Eastern Theater Command, Nanjing, 210002 China; 2 Nanjing Center for Disease Control and Prevention, Nanjing, 210002 China; 3 Chinese Field Epidemiology Training Program, Beijing, 100050 China; 4 Wuxi Center of Joint Logistic Support Force, Wuxi, 214000 China; 5 Department of Epidemiology, College of Preventive Medicine, Army Medical University, Chongqing, 400038 China

**Keywords:** Infectious diseases, Diseases, Health care, Medical research, Risk factors

## Abstract

Many studies have shown that the relationship between ambient temperature, relative humidity and mumps has been highlighted. However, these studies showed inconsistent results. Therefore, the goal of our study is to conduct a meta-analysis to clarify this relationship and to quantify the size of these effects as well as the potential factors. Systematic literature researches on PubMed, Embase.com, Web of Science Core Collection, Cochrane library, Chinese BioMedical Literature Database (CBM) and China National Knowledge Infrastructure (CNKI) were performed up to February 7, 2022 for articles analyzing the relationships between ambient temperature, relative humidity and incidence of mumps. Eligibility assessment and data extraction were conducted independently by two researchers, and meta-analysis was performed to synthesize these data. We also assessed sources of heterogeneity by study region, regional climate, study population. Finally, a total of 14 studies were screened out from 1154 records and identified to estimate the relationship between ambient temperature, relative humidity and incidence of mumps. It was found that per 1 °C increase and decrease in the ambient temperature were significantly associated with increased incidence of mumps with RR of 1.0191 (95% CI: 1.0129–1.0252, *I*^2^ = 92.0%, Egger’s test *P* = 0.001, N = 13) for per 1 °C increase and 1.0244 (95% CI: 1.0130–1.0359, *I*^2^ = 86.6%, Egger’s test *P* = 0.077, N = 9) for per 1 °C decrease. As to relative humidity, only high effect of relative humidity was slightly significant (for per 1 unit increase with RR of 1.0088 (95% CI: 1.0027–1.0150), *I*^2^ = 72.6%, Egger’s test *P* = 0.159, N = 9). Subgroup analysis showed that regional climate with temperate areas may have a higher risk of incidence of mumps than areas with subtropical climate in cold effect of ambient temperature and low effect of relative humidity. In addition, meta-regression analysis showed that regional climate may affect the association between incidence of mumps and cold effect of ambient temperature. Our results suggest ambient temperature could affect the incidence of mumps significantly, of which both hot and cold effect of ambient temperature may increase the incidence of mumps. Further studies are still needed to clarify the relationship between the incidence of mumps and ambient temperature outside of east Asia, and many other meteorological factors. These results of ambient temperature are important for establishing preventive measures on mumps, especially in temperate areas. The policy-makers should pay more attention to ambient temperature changes and take protective measures in advance.

## Introduction

Mumps, also known as epidemic parotitis, which is an acute respiratory infectious disease caused by mumps virus belonging to the Paramyxovirus family, is characterized by unilateral or bilateral swollen and painful parotid gland with precursory fever^1^. Mumps is usually a self-limiting disease, and often occurs in children and adolescents. However, mumps virus can invade other organs and the central nervous system through blood circulation, resulting in up to 10% with severe complications, such as meningoencephalitis, meningitis, orchitis, pancreatitis, ovarian inflammation, and inflammation of many other organs^2^. In general, mumps is transmitted by droplets and direct contact, which are infectious in seven days before and nine days after parotid gland enlargement^3^. Moreover, the susceptible population is children under 15, especially those aged between five and nine years old^4^.

Mumps is an infectious disease that can be prevented effectively by vaccination, which is most often incorporated into national immunization programs in a combined measles-mumps-rubella (MMR) vaccine. Since the mumps vaccine was approved for use in 1967, the incidence of mumps decreased dramatically in countries where large-scale immunization against mumps has been implemented^4,5^. By the end of 2016, a total of 121 countries nationwide belonging to the WHO have adopted the mumps vaccine^6^, and 123 countries by the end of 2020^7^. However, the effect of mumps vaccination is determined by many factors, such as age, coverage, the potential evolution of mumps virus, and inoculation times^8^. Mumps outbreaks have recently reemerged in some areas and countries with high mumps immunization rates, which have caused wide concerns regarding its re-outbreak^9–12^. In most parts of the world, the annual incidence of mumps in the absence of immunization is in the range of 100–1000 cases/100 000 population, with epidemic peaks every two to five years, and natural infection with this virus is thought to confer lifelong protection^13,14^. In China mainland, despite the use of mumps virus (MuV) vaccination since 2008, 194 MuV strains were also isolated from 10 of 31 provinces in China mainland in the MuV virology surveillance during 2013–2015^4^. Therefore, the mumps is still a serious public health issue in China.

Meteorological factors have been considered as important influencing factors to many disease, including non-infectious diseases, such as blood pressure^15^, suicide^16^, stroke occurrence^17^, and infectious diseases, such as hand, foot, and mouth disease (HFMD)^18^, influenza^19,20^, tuberculosis^21–23^ and so on. Many articles have tried to clarify the mechanisms between mumps and meteorological factors. The potential biological mechanisms of the relationship between ambient temperature, humidity and incidence of mumps can be explained by survival and reproduction of pathogens, host population, and environmental factors. Several means may be used by meteorological factors to affect the incidence of infectious disease. First, environmental factors such as warm climate and high humidity may promote the survival of the virus in the humid environment^24^. Then, the host population behavior pattern may be affected by the ambient temperature. Many studies have found that there was significant association between weather conditions and physical activity in outdoor environments^25^, while the physical activity of adolescent would be less in winter and would be more during warm months^26^, thereby increasing the chance of contact with pathogens in warm months^27^. In addition, the low air circulation of indoor environment makes the air-borne infectious disease communicate more easily in winter, especially crowded area, such as school. Many researches have attempted to explore the relationship with meteorological factors, especially ambient temperature and relative humidity^28–30^, but the results are inconsistent quantitatively or qualitatively. Therefore, the aim of this study is to systematically identify and review epidemiological evidence related to ambient temperature, relative humidity and the incidence of mumps, as well as to quantify the size of these effects and to identify potential factors.

## Methods

We carried out this meta-analysis in accordance with the Preferred Reporting Items for Systematic review and Meta-Analysis (PRISMA) statement^31^.

### Search strategy

Systematic research was performed in both English and Chinese database, including PubMed, Embase.com, Web of Science Core Collection, Cochrane library, Chinese Biomedical Literature Database (CBM) and the China National Knowledge Infrastructure (CNKI) for relevant studies up to February 7, 2022. Medical Subject Headings (MeSH) Term and free words strategy were applied to maximize the output. The search strategy, which was referenced to Qiang Cheng et al.^18^, was listed as follows: (“temperature” OR “humidity” OR “rainfall” OR “precipitation” OR “atmospheric pressure” OR “air pressure” OR “barometric pressure” OR “climate” OR “Meteorolog*” OR “weather” OR “wind speed” OR “wind velocity” OR “sunshine duration”) AND (mumps OR epidemic parotitis).

After deleting duplicates, all abstracts and titles were filtered independently by two reviewers to remove the irrelevant articles (T.W. Wang and J.J. Wang). Two authors evaluated potential publications by checking their titles and abstracts and then procured the most relevant publications for further full-text examination. Bibliographies section of retrieved articles were also reviewed for additional pertinent studies that were possibly missed in the initial search. If any agreement can’t be solved by discussion, a third reviewer (J.H. Zhang) was requested to make arbitration.

### Inclusion and exclusion criteria

Studies included for further analysis should fulfill the rules as follows: (i) the outcome measure was incidence of mumps at a monthly, weekly or daily resolution; (ii) the exposure of interest was mean ambient temperature and/or relative humidity; (iii) the estimated parameter was the rate of incidence (RR) of mumps associated with each 1 °C increase/decrease in ambient temperature and each 1% increase/decrease in relative humidity, or which can be converted to standardized RR using the following formula^18,32^: ${\text{RR}}_{{\rm standardised}}={\text{RR}}_{{{{\rm (Original)}}}}^{{{{\rm Increment(1)}}/{{\rm Increment(Original)}}}}$, or studies could provide effect estimates of approximate value, such as the excess risk (ER), percentage change(PC), and then convent to RR using the following rule: ER(%) = (RR−1) × 100%^33^, or RR = 1 + PC^17^; and (iv) provided the corresponding 95% confidence interval(CI); (v) the study incorporated at least 4 year of continuous data and controlled for potential confounding factors.

The exclusion criteria of our literature were listed as follows: (i) the full text of the article could not be found; (ii) the format of the paper is not a research article; (iii) articles do not contain data for meta-analysis; and (iv) duplicate publication, or the data was covered by another study included completely.

### Data extraction

The following information was extracted from the studies that met the inclusion criteria: first author, publication year; location, study period; population, ages, exposure meteorological factor, data sources, measures of exposure (95% CI) and outcome, statistical model and so on.

Due to some studies providing different hysteresis patterns to estimate the delayed effects of ambient temperature and relative humidity^18^, such as single-day hysteresis and cumulative hysteresis^34^, we chose the estimate effect with the following rules. If only one lag estimate was provided (either because only one was analyzed or only one was reported from the study), this estimate was recorded. If multiple lags were reported, we chose one based on the following criteria: (1) the lag that the investigators focused on or stated as a priority, (2) the lag that was statistically significant, (3) the lag with the largest effect estimate^35^.

In addition, if the threshold effect existed in the studies, and two effect values be given, we pooled them separately, such as ambient temperature with hot effect and cold effect. Where multiple estimates were reported, we selected the final model as specified by the authors or, if a final model was not specified, the model with the greatest number of relevant covariates based on the Cochrane Collaboration guidelines^36^. If more than one place were reported in one study, we selected the overall effect or pooled it as one single study to avoid over weight any one study.

### Quality assessment of studies in meta-analysis

A new domain-based risk of bias (RoB) assessment tool was adopted to assess the RoB of included studies, which was developed by experts convened by the WHO^37^. This tool provides a description of the instrument devised to assess the risk of bias in the individual studies included in the systematic reviews of adverse health effects informing the guidelines. A total of 13 items are grouped into six domains, which include confounding, selection bias, exposure assessment, outcome measurement, missing data, and selective reporting in the instrument. Each item could be evaluated as low, moderate, and high risk of bias. The results for each domain were analyzed separately, without considering a single result for the whole article. If only one item of the same domain was judged as having high RoB, the entire domain was classified as having “High Risk”. If all domains in one study were low risk of bias, the study could be classified as “Low Risk” study; if at least two domains were high risk of bias, the study would be classified as “High Risk” study; Otherwise, the study was classified as “Moderate Risk”. Further detailed assessments were referred to in similar articles^38–40^.

### Meta-analysis

For standardized results obtained from each study included, we assessed the heterogeneity of the relationship between ambient temperature, relative humidity and incidence of mumps before got the pooled results^36^. Statistical heterogeneity was evaluated by Cochran’s Q test (with *P* < 0.10 indicating statistically significant heterogeneity) and the *I*^2^ statistic^41^. An *I*^2^ from 0 to 40% was treated as an unimportant heterogeneity, *I*^2^ from 30 to 60% was treated as moderate heterogeneity, *I*^2^ from 50 to 90% was treated as substantial heterogeneity and *I*^2^ from 75 to 100% was treated as considerable heterogeneity^42^. A pooled model was chosen based on the heterogeneity: if obvious heterogeneity existed, a random effects model was adopted; otherwise, a fixed effects model was adopted. In addition, subgroup analysis and meta-regression were also performed to explore the potential source of heterogeneity if obvious heterogeneity was found. Furthermore, sensitivity analysis was performed by two means, (i) by converting the pooled results from a random effects model to a fixed effects model or from a fixed effects model to a random effects model; (ii) “leave-one-out” analyses, which was performed by sequentially removing individual studies and evaluating the effect on the overall estimate. Publication bias was tested by funnel plot, Begg’s and Egger’s test^43^. All statistical tests for the meta-analysis were carried out using R software version 4.1.2^44^ (with the package “meta” version 4.18–0^45^). A *P* value of ≤ 0.05 was considered to indicate statistical significance for all tests and models.

## Results

### Study selection and characteristics

**Figure 1:**
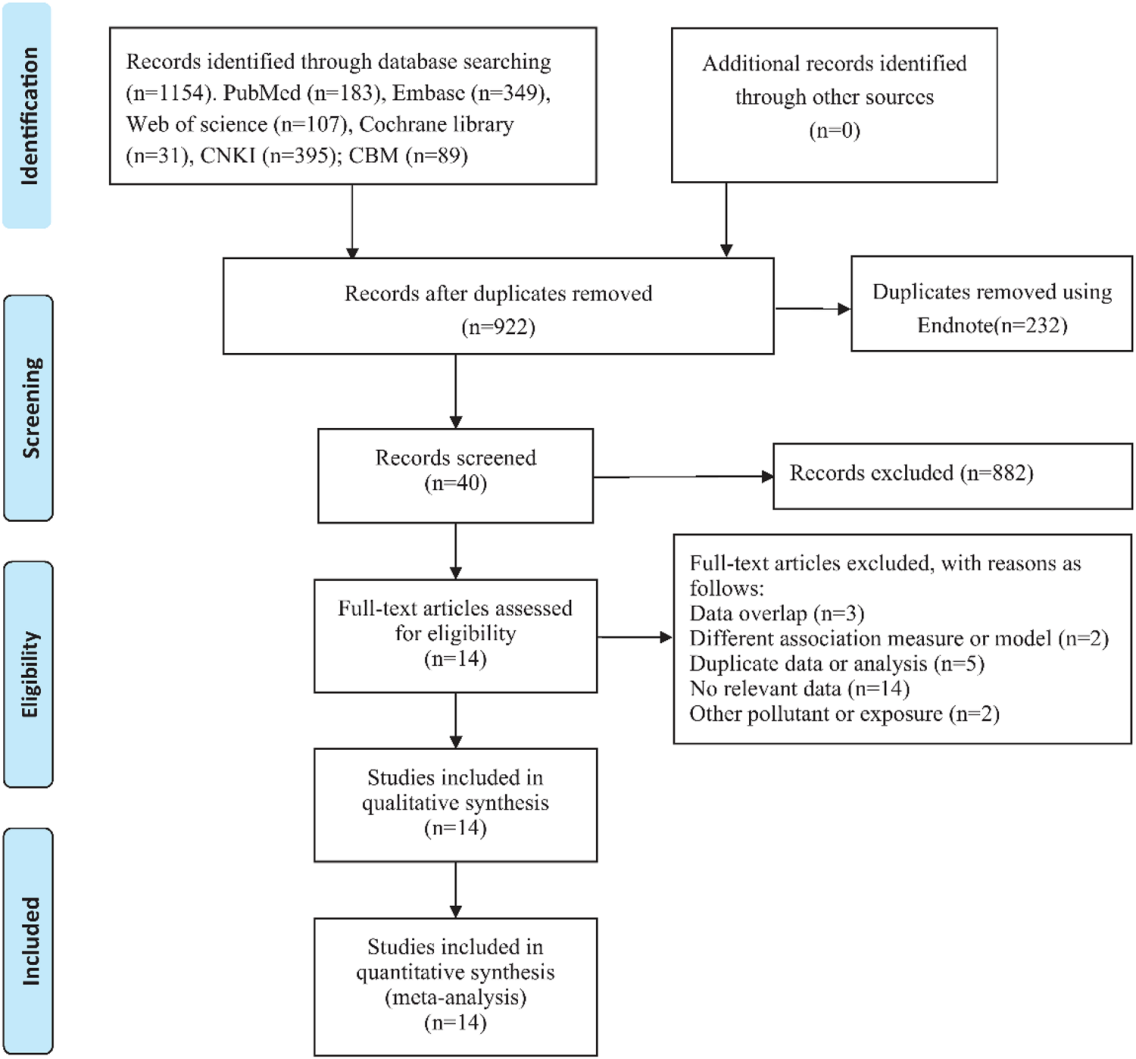
Systematic search and study selection.

A total of 1154 records were obtained through electric searches. After removing duplicates and implementing the inclusion/exclusion criteria, 14 studies were finally included for further study^28–30,46–56^ (Fig. 1). In this paper, there are 14 studies on the relationship between ambient temperature and incidence of mumps (13 for hot effect and 9 for cold effect), and 11 studies on the relationship between relative humidity and incidence of mumps (9 for high relative humidity and 7 for low relative humidity). Of all studies included, 11 were from Chinese mainland^28,30,46,47,49–55^, 2 from China Taiwan^48,56^, 1 from Japan Fukuoka^29^. The characteristics of the studies included were shown in Table 1.

**Table 1:** Basic characteristics of the studies on the relationships between ambient temperature and relative humidity with mumps.

First Author	Year	Study Location	Study Period	Exposure variable	Data source	Outcome	Effect index	Statistical model	Temporal lags	Resolution	Population	Ratio of M/F	Age
Lin ChengYao	2021	Taiwan, China	2012–2018	Mean temperature, relative humidity	CDC	Reported cases	ER	Poisson regression models	not mentioned	Monthly	5,459	3192/2267	All and 1 ~ 19 years old accounted for 66%
Zeng Weilin	2021	Four city of Guanggong, China	2005–2018	Mean temperature;	CDC	Reported cases	RR	DLNM*	30	Daily	212,109	not mentioned	all
Lin Shaoqian	2021	Jinan, China	2014–2018	Mean temperature;	CDC	Reported cases	ER	GAM	3	Weekly	4141	2559/1582	0–14 years old accounted for 81.09%
Dandan Zhang	2020	Shandong, China	2009–2017	Mean temperature; relative humidity;	CDC	Reported cases	RR	DLNM	30	Daily	104,685	1.91:11	ALL
Zonghui Fan	2020	Jiayuguan, China	2008–2016	Mean temperature; relative humidity;	CDC	Reported cases	RR	DLNM	14	Daily	1400	1.47∶1	6–14 years old accounted for 60.36% (845)
Wu Huabing	2020	Hefei, China	2011–2016	Mean temperature; relative humidity;	CDC	Reported cases	RR	GAM and DLNM	30	Daily	9676	1.86:1	–
Jianyun Lu	2020	Guangzhou, China	2014–2018	Mean temperature; relative humidity;	CDC	Reported cases	RR	DLNM	21	Daily	9842	1.83:1	Children (younger than 18)
Tian Liu	2019	Jingzhou, China	2010–2017	Mean temperature; relative humidity;	CDC	Reported cases	RR	DLNM	30	Daily	8252	1.71:1	0–14 years old accounted for 88.56%
Sheng Li	2018	Lanzhou, China	2008–2016	Mean temperature;	CDC	Reported cases	RR	DLNM	14	Daily	11,762	1.52∶1	6–14 years old accounted for 49.67% (5843)
Yu Guoqi	2018	Guangxi, China	2005–2017	Mean temperature; relative humidity;	CDC	Reported cases	RR	DLNM and meta	30	Daily	183,341	Not mentioned	–
Hu Wenqi	2018	Fujian, China	2005–2013	Mean temperature; relative humidity;	CDC	Reported cases	RR	Quasi-Poisson GAM and DLNMs	30	Daily	75,249	1.82:1	Children aged 5–9 accounting for 39.96%
Yang Qiongying	2014	Guangzhou, China	2005–2012	Mean temperature; relative humidity;	CDC	Reported cases	RR	DLNM	30	Daily	49,760	1.75∶1	0–14 years old accounted for 81.59%
Yi-Chien Ho	2015	Taiwan, China	2006–2011	Mean temperature; relative humidity;	Taiwan CDC	Reported cases	IRR	Poisson regression models	not mentioned	Weekly	6612	61.7:38.3	0–10 years old accounted for 50.9%
Onozuka, D	2011	Fukuoka, Japan	2000–2008	Mean temperature; relative humidity;	*	Reported cases	PC	Negative binomial regression	2	Weekly	67,000	not mentioned	All below 14

DLNM, Distributed Lag Non-Linear Models; RR, Relative Risk; PC, Percentage Change; ER, Excess Risk; IRR, Incidence Rates Ratio; GAM, Generalized Additive Model.

*:120 sentinel medical institutions within Fukuoka Prefecture.

### Risk of bias assessment

**Figure 2:**
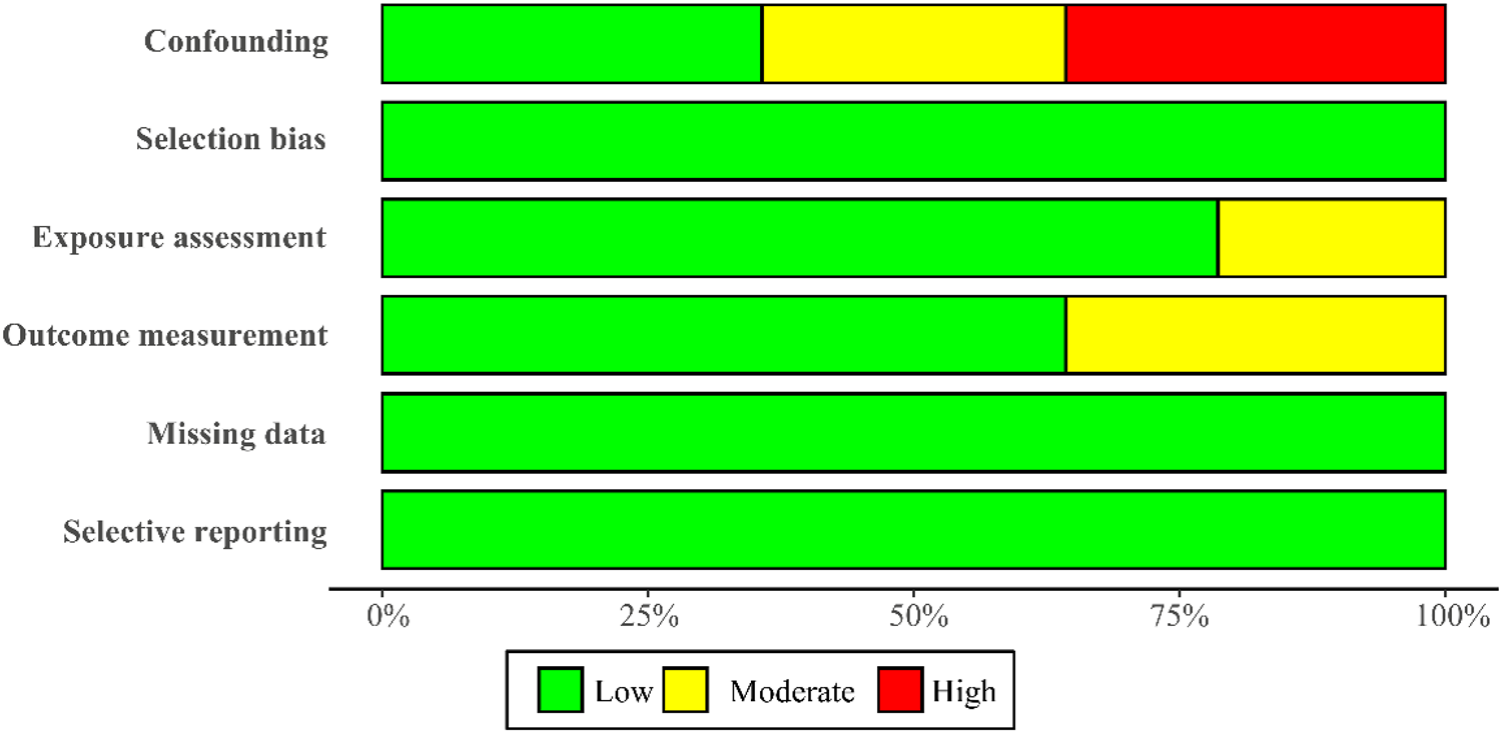
Summary of the risk of bias assessment.

The summary of the risk of bias assessment is shown in summary plot (Fig. 2). In three out of six domains (selection bias, missing data, selective reporting), the risk of bias was found to be only low, and the risk of bias of two domains (exposure assessment, outcome measurement) was found to be only low or moderate. But in the domain of confounding, we found a variable proportion of articles having high and moderate risk of bias, of which 35.7%(5) articles were classified as high risk of bias on confounding. The main reason for the high risk of bias in the confounding domain was the lack of adjusting critical confounders (seasonality, long-term trends, day of the week) and/or additional confounders (holidays). Based on the rules of the RoB assessment for a study, five studies were classified as “Moderate Risk”, and the rests were classified as “Low Risk”. In this study, especially for studies conducted in China mainland, the data of exposure assessment and outcome measurement were mainly from the local Center for Disease Control and Prevention, and the local Meteorological Bureau. However, some studies didn’t clearly state how to collect and manage the data, and define the cases, which made two domains of exposure assessment and outcome measurement of some studies were classified as “Moderate Risk”. In total, based on our assessment results, the quality of included studies was medium to high quality.

### Overall effects analysis

**Figure 3:**
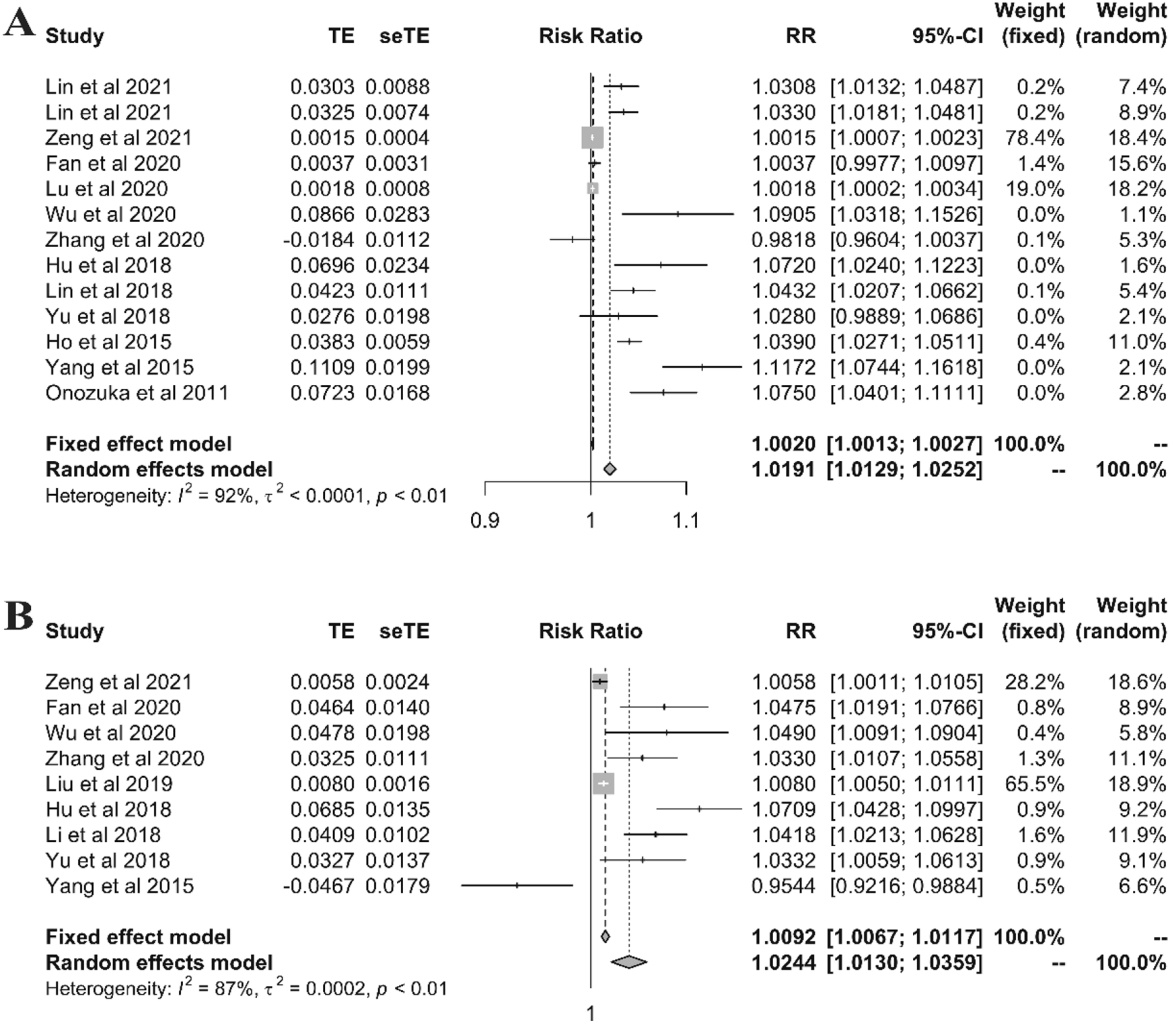
Meta-analysis of hot effect and cold effect of ambient temperature and incidence of mumps. (**A** for hot effect, **B** for cold effect).

**Figure 4:**
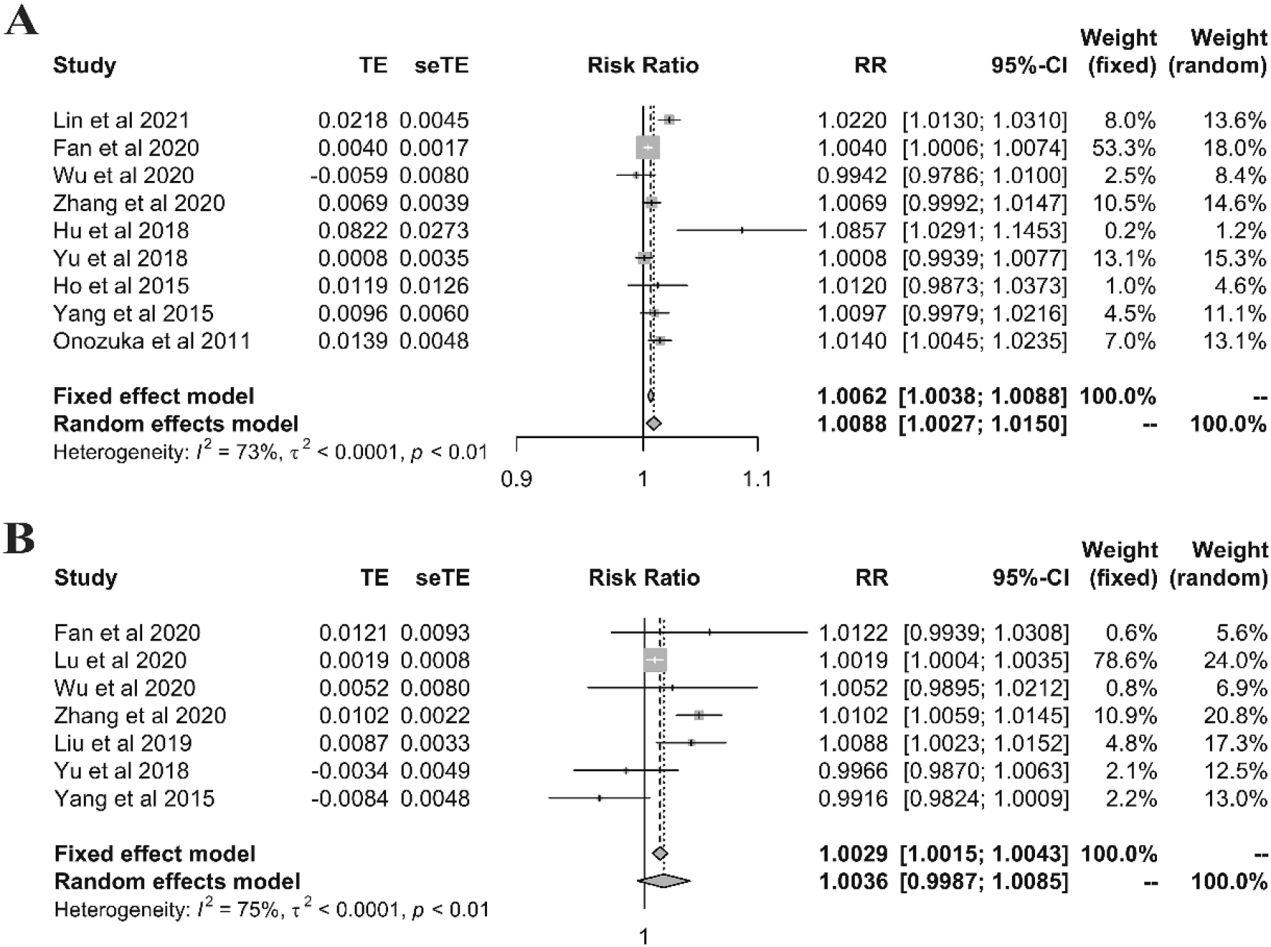
Meta-analysis of high and low effect of relative humidity and incidence of mumps. (**A** for high relative humidity, **B** for low relative humidity).

As the effects expressed in original studies, both ambient temperature and relative humidity showed high and low effect, hot/cold effect in ambient temperature and high/low values in relative humidity. The sizes of the effects obtained from each dataset are presented in the forest plots along with the overall results of the meta-analysis (Figs. 3 and 4). The pooled results of meta-analysis with 14 studies indicated that per 1 °C increase and decrease in the ambient temperature were statistically associated with incidence of mumps, with RR(95% CI) of 1.0191 (1.0129–1.0252) for per 1 °C increase (Fig. 3A) and 1.0244 (1.0130–1.0359) for per 1 °C decrease(Fig. 3B). At the same time, for per 1 unit change of relative humidity, high relative humidity was significant (1.0088 (1.0027–1.0150)), while low relative humidity not (1.0036 (0.9987–1.0085)). Nevertheless, significant heterogeneity was also observed between the studies in both ambient temperature and relative humidity (Figs. 3 and 4), indicating that subgroup analysis was necessary.

### Subgroup and meta-regression analyses

We conducted subgroup analysis based on the following variables that we could obtain to explore the potential source of heterogeneity: study region (Province level vs City level), regional climate (Subtropical vs Temperate), study population (All vs Children only).

As shown in Tables 2 and 3, of the three factors we explored, only two subgroups showed statistically significant association with mumps, that were regional climate in cold effect of ambient temperature and low relative humidity. In the cold effect of ambient temperature, people lived in temperate region had higher relative risk than in subtropical region along with decrease of ambient temperature. Similar situation happened in low relative humidity effect, the relative risk was significantly higher in temperate region along with the decrease of relative humidity, while no significant difference was found in subtropical region.

**Table 2:** Subgroup analysis of the incidence of mumps with ambient temperature.

Effect	Subgroup types	Studies (n)	Pooled	Heterogeneity		Between-group differences (Q/*P* value)
			RR (95% CI)	*I*^2^ (%)	*P* value	
Hot effect	Region level					1.09/0.2976
	Province	6	1.0219 [1.0007; 1.0436]	92.8	< 0.01	
	City	7	1.0362 [1.0203; 1.0524]	92.4	< 0.01	
	Regional climate					0.19/0.6642
	Subtropical	8	1.0184 [1.0114; 1.0255]	93.3	< 0.01	
	Temperate	5	1.0240 [1.0000; 1.0485]	89.4	< 0.01	
	Population					0.02/0.8771
	All	11	1.0300 [1.0168; 1.0433]	92.4	< 0.01	
	Children only	2	1.0357 [0.9666; 1.1097]	94.3	< 0.01	
Cold effect	Region level					0.92/0.3364
	Province	4	1.0375 [1.0047; 1.0714]	90.4	< 0.01	
	City	5	1.0176 [0.9943; 1.0414]	85.9	< 0.01	
	Regional climate					9.28/0.0023
	Subtropical	5	1.0134 [1.0014; 1.0257]	88.0	< 0.01	
	Temperate	4	1.0409 [1.0281; 1.0537]	0	0

**Table 3:** Subgroup analysis of the incidence of mumps with relative humidity.

Effect	Subgroup types	Studies (n)	Pooled	Heterogeneity		Between-group differences (Q/*P* value)
			RR (95% CI)	*I*^2^ (%)	*P* value	
High relative humidity	Region level					0.96/0.3261
	Province	5	1.0132 [1.0010; 1.0255]	81.0	< 0.01	
	City	4	1.0050 [1.0020; 1.0081]	51.9	0.10	
	Regional climate					0.35/0.5530
	Subtropical	6	1.0110 [0.9988; 1.0233]	79.3	< 0.01	
	Temperate	3	1.0054 [1.0024; 1.0084]	50.0	0.14	
	Population					1.01/0.3157
	All	8	1.0081 [1.0014; 1.0148]	73.5	< 0.01	
	Children only	1	1.0140 [1.0045; 1.0235]	–	–	
Low relative humidity	Region level					0.03/0.8643
	Province	2	1.0041 [0.9909; 1.0175]	84.2	0.01	
	City	5	1.0028 [0.9973; 1.0084]	61.2	0.04	
	Regional climate					7.34/0.0068
	Subtropical	5	1.0012 [0.9962; 1.0063]	61.4	0.03	
	Temperate	2	1.0103 [1.0061; 1.0145]	0.0	0.84	
	Population					0.31/0.5800
	All	6	1.0039 [0.9971; 1.0107]	71.5	< 0.01	
	Children only	1	1.0019 [1.0004; 1.0035]	–	–	

To better understand the influence of factors, we also performed meta-regression analysis based on the information we obtained. As shown in Table 4, we found that regional climate could affect the association between hot effect of ambient temperature and incidence of mumps, which was also consistent with the results of subgroup analysis.

**Table 4:** Meta-regression analysis of mumps on ambient temperature and relative humidity.

Index	Effect	Factors	Estimate	se	zval	*P* value	ci.lb	ci.ub
Ambient temperature	Hot effect	Region level	−0.0430	0.0218	−1.9764	0.0481	−0.0856	−0.0004
		Regional climate	−0.0345	0.0201	−1.7146	0.0864	−0.0739	0.0049
		Population	−0.0250	0.0262	−0.9515	0.3413	−0.0764	0.0265
	Cold effect	Region level	0.0318	0.0188	1.6929	0.0905	−0.0050	0.0686
		Regional climate	0.0381	0.0189	2.0114	**0.0443**	0.0010	0.0752
Relative humidity	High effect	Region level	0.0094	0.0094	0.9994	0.3176	−0.0090	0.0278
		Regional climate	−0.0038	0.0099	−0.3822	0.7023	−0.0231	0.0156
		Population	0.0130	0.0150	0.8674	0.3857	−0.0164	0.0424
	Low effect	Region level	−0.0041	0.0083	−0.4959	0.6200	−0.0205	0.0122
		Regional climate	0.0121	0.0087	1.3831	0.1666	−0.0050	0.0292
		Population	0.0004	0.0090	0.0406	0.9676	−0.0172	0.0179

### Sensitivity analysis

Two types of sensitivity analyses were performed. Firstly, we removed any single study to verify whether the overall effect was affected. The results showed that no significant difference was found except for the effect of relative humidity. After removing the study of Yang^55^, the result become significant with RR (95%) of 1.0054 (1.0005, 1.0103) in low relative humidity. Secondly, sensitivity analysis was performed by changing the pooled results by converting the pooled model (from random effects model to fixed effects model). The results exhibited no big differences before and after pooling of ambient temperature effect, indicating stability in the pooled results. However, similar results were not found in the low effect of relative humidity. Overall, the results of low effects of relative humidity were not stable.

### Publication bias

**Figure 5:**
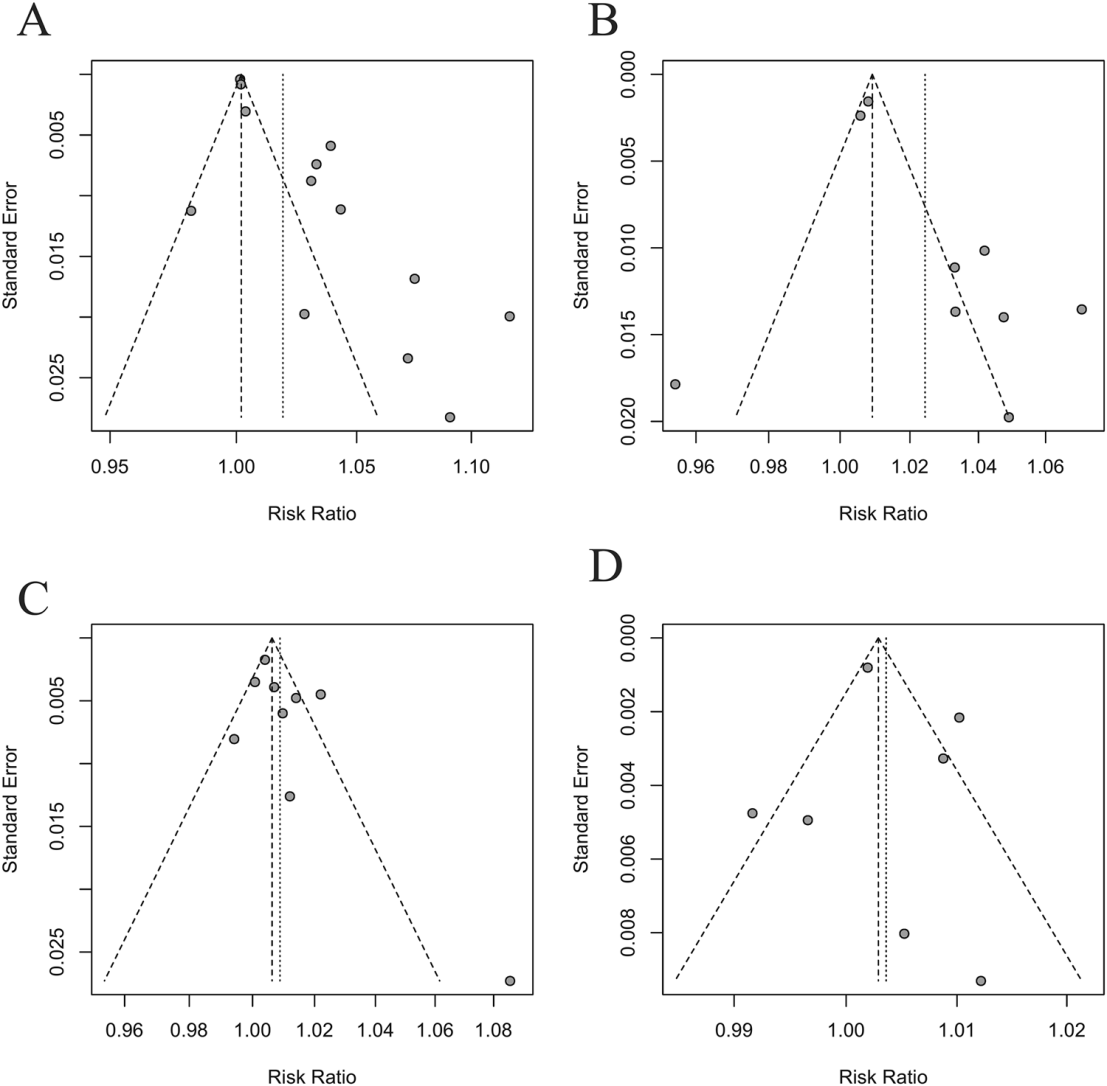
Funnel plot of the association between ambient temperature, relative humidity and incidence of mumps. (**A** hot effect of ambient temperature; **B** cold effect of ambient temperature; **C** high effect of relative humidity; **D** low effect of relative humidity).

We plotted the funnel plot and performed Begg’s and Egger’s tests to check whether publication bias exists. As shown in Fig. 5 and Table 5, publication bias may exist in ambient temperature, especially in hot effect of ambient temperature.

**Table 5:** Publication bias test of temperature and relative humidity.

Effect	No. of studies included	Method	Statistics	*P* value
Hot effect of ambient temperature	13	Egger’s test	4.59	0.001
		Begg's test	1.10	0.272
Cold effect of ambient temperature	9	Egger’s test	2.07	0.077
		Begg's test	0.00	1.000
High effect of relative humidity	9	Egger’s test	1.57	0.159
		Begg's test	1.46	0.144
Low effect of relative humidity	7*	Egger’s test	0.39	0.711
		Begg's test	0.45	0.652

*As the numbers of studies for Egger's and Begg's tests are suggested to be more than 10, the results of some groups may be biased.

## Discussion

Although vaccination was widely used in mumps prevention, dramatic increases in mumps cases have received much attention in recent years^57^. China, as one of the countries including the MMR into the immunization programs, also has a relative high morbidity of mumps among adolescents^58^. To date, this study was the first meta-analysis to examine the relationship between ambient temperature, relative humidity and incidence of mumps. In the 14 articles included in this paper, we identified significant positive relationship between ambient temperature and incidence of mumps, and high relative humidity, while no relationship was found between low relative humidity and incidence of mumps. We also found that there was also significant heterogeneity in the estimated association, and subgroup analysis also showed that people living in temperate regions had a higher risk of incidence of mumps than in subtropical regions in cold effect of ambient temperature and low effect of relative humidity, which was also confirmed by meta-regression in cold effect of ambient temperature.

To our surprise, relative humidity is found to be slightly associated with mumps in our study, only high relative humidity with RR of 1.0088 (1.0027–1.0150). In previous study, humidity was found to be associated with many diseases, such as hand, foot, and mouth disease^59,60^, dengue outbreaks^61^, COVID-19^62^, et al. Many reasons may be used to explain this phenomenon. First, compared with the other pathogen, the pathogenicity of mumps virus may have different effect on humidity. The effect of mumps may not be affected by humidity significantly. Second, as to humidity, the publication bias was found, leading to that the pooled results may be affected by unpublished studies. Third, obvious heterogeneity also existed. Compared with ambient temperature, the effect of humidity is relative smaller per 1 unit, and could be affected by many factors. Fourth, even some factors were adjusted, such as school holidays, long-term trend, and seasonality, the confounding factor may not be fully explained, and the number of studies included for humidity is not large enough, only 11 studies. In addition, sensitivity analysis showed that the results of relative humidity were not stable. Therefore, the results may be unstable, indicating further exploring may be still needed.

In detail, the ambient temperature affected the incidence of mumps in both hot and cold effect, indicating hotter and colder ambient temperature had higher risk than the thresholds, which was corresponding to the fact that non-linear relationships of meteorological factors with morbidity and mortality^47^. The pathogen of mumps has clear tolerance of ambient temperature in biological mechanism, which had bad tolerance of high ambient temperature and relative better tolerance of low tolerance. However, as the difference in environmental and climate factors, which led to the different thresholds of meteorological factors, such as threshold of ambient temperature in our study, ranging from about 10 °C and 25  °C, mainly focusing on 20 °C to 25 °C, which may also contribute to the heterogeneity. What's more, the non-linear relationships were also complex. In our studies included, several types were found, such as inverse “S”^46^, inverse “V”^49,51^, “V”^50^, and even “M”^50^, et al. However, in our study, based on the information provided in the included studies, we only obtained the main part result, such as only “V” part of “M” shape.

Previous studies indicated a slightly lower pooled value and narrower confidence interval than the studies of weekly resolution, suggesting it may be more reasonable to use the daily exposure measures in future research designs, especially for those diseases with shorter incubation period^18^. In our studies, 12 studies reported cases with resolution of daily, 3 of weekly and 1 of monthly. As only 3 with weekly data and 1 with monthly data, we didn’t perform subgroup analysis based on time resolution. As mumps is an acute infectious disease, research based on daily data may be more suitable for disease control and prevention by providing more timely information. In the future, similar studies may perform sensitivity analysis by changing the resolution to confirm the effect of resolution and the stability of overall and specific results.

In our subgroup analysis, only the regional climate was found to affect the relationship between meteorological factors and mumps. Human activities may be one of the most important reasons. Temperate climate may be more suitable for outdoor activities^25^, especially for children and adolescents. Mumps is a disease mainly happens in younger people, which also increase the risk for children and adolescents from temperate region. It is reported that a total of about 500 thousand cases was reported in 2018^63^, and half of them happened in China^64^. However, in our study, all studies are from Chinese mainland, China Taiwan and Japan, which may not represent other places, such as tropical and frigid. Therefore, researchers may pay more attention to other climate regions in the future.

Distributed Lag Non-linear Models, which was achieved with “dlnm” package constructed by Gasparrini A^65^ within the statistical environment R, provided much convenience for analysis between meteorological factors and disease. In the meta-analysis, most of include studies were performed with this package. However, due to complexity between meteorological factors and disease, and no standard report guideline, all of these led to the difficulty for meta-analysis. To solve this situation, the author has built a package “mvmeta” within the statistical environment R to pool the results from multiple cities^66–68^. However, this two-stage analysis is based on the individual data, which is hard to obtain for meta-analysis. Therefore, if the authors provided the RR (or OR, PC, ER et al.) per 1 unit in each line trend range in both single lag day and accumulative lagged effect, meta-analysis of this type analysis may be easier in the future.

Although this was the first meta-analysis with 16 studies to examine the relationship between ambient temperature, relative humidity and incidence of mumps, our meta-analysis has several limitations that should be recognized when interpreting the results. Firstly, the heterogeneity is obvious. Although we have performed subgroup and meta-regression analysis, and found the great importance of climate, obvious heterogeneity still existed. In addition, when available, adjusted estimates were used in preference to unadjusted estimates. Even though the adjusted estimates may be closer to the true effect for adjusted results could control confounding factors, the different adjusted factors in different studies may also contribute to the heterogeneity. Secondly, publication bias may exist in both ambient temperature and relative humidity. Researchers were more trend to publish statistically significant findings, which may lead to the ignorance of negative results. Thirdly, all studies for meta-analysis were from East Asian (Chinese mainland, China Taiwan and Japan), with limited adjustment for confounders, which may limit the extension to other places. Fourthly, most of the included studies tend to report the largest effect estimates, and the effect estimates of different lag days is not fully available for analysis, we ignored the lag effect and chose the largest effect estimates for meta-analysis, which also contributed to heterogeneity. Fifthly, although our study was performed with strict inclusion and exclusion criteria, a total of 14 studies were excluded for no relevant data, which may also affect our results. Sixthly, in China, the mumps vaccine has been used since 2008 and its efficacy is a function of age, coverage, and timing of the inoculation. All Chinese studies included were conducted after 2008. Thus, even by controlling for confounding, it is very difficult to disentangle the contribution of meteorological factors from vaccination efficacy on mumps incidence. However, as the widely use of mumps vaccine, our results may be more suitable for current preventive measures. Finally, different confounding factors were adjusted in different studies, which may also lead to heterogeneity and bias of risk. Nevertheless, our study is still the most comprehensive about the association between incidence of mumps and ambient temperature/relative humidity.

## Conclusion

Overall, the pooled results of our meta-analysis provide systematic evidence that ambient temperature may increase the incidence of mumps, especially in east Asia regions. As limitation of the number of studies for relative humidity and the significant effect of relative humidity on other air-borne diseases, the effect of relative humidity on mumps needs further exploring. Further studies are still needed to clarify the relationship between ambient temperature and mumps in areas outside of east Asia regions, and many other meteorological factors. These results of ambient temperature are important for establishing preventive measures on mumps, especially in temperate regions.

## Supplementary Information

Supplementary Information 1.

Supplementary Information 2.

## Data Availability

Only aggregated summaries of the data are provided in this manuscript. However, all data generated in this study can be made publicly available on request. Please contact the corresponding author for any kind of data request.
