# Supplementary material for: Meta-analysis of the effects of ambient temperature and relative humidity on the risk of mumps
Source: Sci Rep. 2022 Apr 19;12:6440. doi: 10.1038/s41598-022-10138-7 (PMC9017417; doi:10.1038/s41598-022-10138-7)
Supplement: Supplementary file 1 — Supplementary Information 1. [file 41598_2022_10138_MOESM1_ESM.doc]

**Search strategy for the relationship between ambient temperature, relative humidity and incidence of mumps**

Search date: February 7, 2022

**Search strategy for PubMed**

**1.Words for ambient temperature, relative humidity (1,145,736 records were found)**

"temperature" OR "humidity" OR "rainfall" OR "precipitation" OR "atmospheric pressure" OR "air pressure" OR "barometric pressure" OR "climate" OR "Meteorolog*" OR "weather" OR "wind speed" OR "wind velocity" OR "sunshine duration"

**2.Words for mumps (11,773 records were found)**

mumps OR "epidemic parotitis"

**3.Combined (183 records were found)**

(mumps OR "epidemic parotitis") AND ("temperature" OR "humidity" OR "rainfall" OR "precipitation" OR "atmospheric pressure" OR "air pressure" OR "barometric pressure" OR "climate" OR "Meteorolog*" OR "weather" OR "wind speed" OR "wind velocity" OR "sunshine duration")

**Search strategy for Embase.com**

**1.Words for ambient temperature, relative humidity (1,455,101 records were found)**

#1 'temperature'/exp OR temperature OR 'humidity'/exp OR humidity OR 'rainfall'/exp OR rainfall OR 'precipitation'/exp OR precipitation OR 'atmospheric pressure'/exp OR 'atmospheric pressure' OR 'air pressure'/exp OR 'air pressure' OR (('air'/exp OR air) AND ('pressure'/exp OR pressure)) OR 'barometric pressure'/exp OR 'barometric pressure' OR (barometric AND ('pressure'/exp OR pressure)) OR 'climate'/exp OR climate OR meteorolog* OR 'weather'/exp OR weather OR 'wind speed'/exp OR 'wind speed' OR (('wind'/exp OR wind) AND ('speed'/exp OR speed)) OR 'wind velocity'/exp OR 'wind velocity' OR (('wind'/exp OR wind) AND ('velocity'/exp OR velocity)) OR 'sunshine duration'/exp OR 'sunshine duration' OR (('sunshine'/exp OR sunshine) AND ('duration'/exp OR duration))

**2.Words for mumps** **(19,554 records were found)**

#2 'mumps'/exp OR mumps OR 'epidemic parotitis'/exp OR 'epidemic parotitis' OR (('epidemic'/exp OR epidemic) AND ('parotitis'/exp OR parotitis))

**3.Combined (349 were records found)**

#3 #1 AND #2

**Search strategy for Web of Science** **Core Collection**

**(107 records were found)**

(temperature OR humidity OR rainfall OR precipitation OR (atmospheric AND pressure) OR (air AND pressure) OR (barometric AND pressure) OR climate OR meteorolog* OR weather OR (wind AND speed) OR (wind AND velocity) OR (sunshine AND duration)) AND (mumps OR (epidemic AND parotitis))

**Search strategy for Cochrane library**

**(31 records were found)**

(temperature OR humidity OR rainfall OR precipitation OR (atmospheric AND pressure) OR (air AND pressure) OR (barometric AND pressure) OR climate OR meteorolog* OR weather OR (wind AND speed) OR (wind AND velocity) OR (sunshine AND duration)) AND ((epidemic parotitis) OR mumps)

**China National Knowledge Infrastructure (CNKI) (old edition)**

**(395 records were found)**

(((((((((((SU=温度) OR (KY=温度) OR (TI=温度) OR (TKA=温度)) OR ((SU='湿度') OR (KY=湿度) OR (TI=湿度) OR (TKA=湿度))) OR ((SU=降水) OR (KY=降水) OR (TI=降水) OR (TKA=降水))) OR ((SU=气压) OR (KY=气压) OR (TI=气压) OR (TKA=气压))) OR ((SU=气候) OR (KY=气候) OR (TI=气候) OR (TKA=气候))) OR ((SU=气象) OR (KY=气象) OR (TI=气象) OR (TKA=气象))) OR ((SU=天气) OR (KY=天气) OR (TI=天气) OR (TKA=天气))) OR ((SU=风速) OR (KY=风速) OR (TI=风速) OR (TKA=风速))) OR ((SU=日照) OR (KY=日照) OR (TI=日照) OR (TKA=日照))) AND (((SU=流行性腮腺炎) OR (KY=流行性腮腺炎) OR (TI=流行性腮腺炎) OR (TKA=流行性腮腺炎)) OR ((SU=腮腺炎) OR (KY=腮腺炎) OR (TI=腮腺炎) OR (TKA=腮腺炎))))

**Chinese Biomedical Literature Database (CBM)**

**(89 records were found)**

("流行性腮腺炎"[全部字段:智能] OR "腮腺炎"[全部字段:智能]) AND ("温度"[全部字段:智能] OR "湿度"[全部字段:智能] OR "降水"[全部字段:智能] OR "降雨"[全部字段:智能] OR "气压"[全部字段:智能] OR "气象"[全部字段:智能] OR "天气"[全部字段:智能] OR "风速"[全部字段:智能] OR "日照"[全部字段:智能])
