# Supplementary material for: Meta-analysis of the effects of ambient temperature and relative humidity on the risk of mumps
Source: Sci Rep. 2022 Apr 19;12:6440. doi: 10.1038/s41598-022-10138-7 (PMC9017417; doi:10.1038/s41598-022-10138-7)
Supplement: Supplementary file 2 — Supplementary Information 2. [file 41598_2022_10138_MOESM2_ESM.docx]

Excluded studies at the full-text review stage

| Index | authors | Year of Publication | Title of the article | Journal | Reasons for exclusion |
| --- | --- | --- | --- | --- | --- |
| 1 | Zha WT et al^1^ | 2020 | Effects of meteorological factors on the incidence of mumps and models for prediction, China | BMC Infectious Diseases | Different association measure or model |
| 2 | Zhou ZF et al^2^ | 2020 | Nonlinear effect of wind velocity on mumps in Shenzhen | Public Health | Other pollutant or exposure |
| 3 | Zhang D et al^3^ | 2019 | The relationship between meteorological factors and mumps based on Boosted regression tree model | Science of the Total Environment | Different association measure or model |
| 4 | Hao J et al^4^ | 2019 | The association between short-term exposure to ambient air pollution and the incidence of mumps in Wuhan | Environmental Research | Other pollutant or exposure |
| 5 | Zhu H et al^5^ | 2019 | Epidemiological Characteristics and Spatiotemporal Analysis of Mumps from 2004 to 2018 in Chongqing | International Journal of Environmental Research and Public Health | No relevant data |
| 6 | Ye Wenjing et al^6^ | 2019 | Analysis of the Association between Intestinal Infectious Diseases and Climate Factors of Fujian Province in 2006-2015 | Journal of Medical Theory and Practice | No relevant data |
| 7 | Hao Y et al^7^ | 2019 | Time series analysis of mumps and meteorological factors in Beijing | BMC Infectious Diseases | No relevant data |
| 8 | Zhang Dandan et al^8^ | 2018 | Effect of temperature on the incidence of mumps in Heze City and Weihai City | Journal Of Shandong University (Health Sciences) | Duplicate data or analysis |
| 9 | Yu GQ et al^9^ | 2018 | Spatial, temporal, and spatiotemporal analysis of mumps in Guangxi Province, China, 2005-2016 | BMC Infectious Diseases | No relevant data |
| 10 | Dai Qiuzhi^10^ | 2018 | Epidemiological Analysis of Measles in Rizhao from 2013 to 2016 | Master thesis of Qingdao University | No relevant data |
| 11 | Zhang Zhi et al^11^ | 2017 | Relation Between Abnormal Epidemic Mumps and Atmospheric Circulation in Yinchuan | Journal of Arid Meteorology | No relevant data |
| 12 | Runzi L et al^12^ | 2016 | The short-term association between meteorological factors and mumps in Jining | Science of the Total Environment | Data overlap |
| 13 | Li Runzi et al^13^ | 2016 | Association between incidence of mumps and meteorological factors in Ji'ning | Journal of Environment and Health | Duplicate data or analysis |
| 14 | Wang Li^14^ | 2016 | Discussion on the relationship between weather conditions and mumps in Zhangjiajie city | Statistics and Management | No relevant data |
| 15 | Lei Jing et al^15^ | 2015 | Analysis of relationship between mumps and meteorological conditions in Yinchuan city | Journal of Ningxia Medical University | No relevant data |
| 16 | Xun Huanmiao^16^ | 2015 | Impacts of Tropical Cyclones on Infectious Diseases in Guangdong Province, 2005-2011 | Master thesis of Shandong University | No relevant data |
| 17 | Zhang Zhi et al^17^ | 2015 | The Epidemic Characteristics of Mumps and Relations with Meteorological Factors in Yinchuan | Journal of Arid Meteorology | No relevant data |
| 18 | Yang Q et al^18^ | 2014 | The relationship between meteorological factors and mumps incidence in Guangzhou | Hum Vaccin Immunother | Duplicate data or analysis |
| 19 | Fu Chuanxi et al^19^ | 2014 | Time series study of relationship between meteorological factors and mumps incidence | South China J Prev Med | Duplicate data or analysis |
| 20 | Wei Xiaojuan et al^20^ | 2014 | Correlation study and prediction model analysis of key infectious diseases and climate in Linyi City | Journal of Community Medicine | No relevant data |
| 21 | Zhang Xuan et al^21^ | 2014 | Correlation between meteorological factors and mumps incidence in Beijing from 1990 to 2004 | Chinese Journal of Traditional Chinese Medicine and Pharmacy | No relevant data |
| 22 | Su Baihua^22^ | 2013 | Correlation between mumps infection and climatic factors in Taiwan | Master thesis of Cheng Kung University | Duplicate data or analysis |
| 23 | Jiang Shaoping et al^23^ | 2013 | Relationship between mumps and climatic factors in Yinchuan city | Journal of Environment and Health | No relevant data |
| 24 | Yang Chao et al^24^ | 2011 | Study on Cold Area of Meteorological Factors to the Impact Ofurban Main Infectious Diseases Paleolimnologic | Chinese Primary Health Care | No relevant data |

**Reference**

1 Zha, W. T. *et al.* Effects of meteorological factors on the incidence of mumps and models for prediction, China. *BMC infectious diseases* **20**, 468, doi:10.1186/s12879-020-05180-7 (2020).

2 Zhou, Z. F. *et al.* Nonlinear effect of wind velocity on mumps in Shenzhen, China, 2013-2016. *Public health* **179**, 178-185 (2020).

3 Zhang, D. *et al.* The relationship between meteorological factors and mumps based on Boosted regression tree model. *Science of the Total Environment* **695** (2019).

4 Hao, J. *et al.* The association between short-term exposure to ambient air pollution and the incidence of mumps in Wuhan, China: A time-series study. *Environmental research* **177**, doi:10.1016/j.envres.2019.108660 (2019).

5 Zhu, H. *et al.* Epidemiological Characteristics and Spatiotemporal Analysis of Mumps from 2004 to 2018 in Chongqing, China. *International journal of environmental research and public health* **16** (2019).

6 Wenjing, Y. *et al.* Analysis of the Association between Intestinal Infectious Diseases and Climate Factors of Fujian Province in 2006-2015. *Journal of Medical Theory and Practice* **32** (2019).

7 Hao, Y. *et al.* Time series analysis of mumps and meteorological factors in Beijing, China. *BMC infectious diseases* **19** (2019).

8 Dandan, Z. *et al.* Effect of temperature on the incidence of mumps in Heze City and Weihai City. *Journal Of Shandong University (Health Sciences)* **56** (2018).

9 Yu, G. Q. *et al.* Spatial, temporal, and spatiotemporal analysis of mumps in Guangxi Province, China, 2005-2016. *BMC infectious diseases* **18** (2018).

10 Dai, Q. *Epidemiological Analysis of Measles in Rizhao from 2013 to 2016* Master thesis, Qingdao University, (2018).

11 Zhi, Z. & Wei, C. Relation Between Abnormal Epidemic Mumps and Atmospheric Circulation in Yinchuan. *Journal of Arid Meteorology* **35**, 116-121 (2017).

12 Runzi, L. *et al.* The short-term association between meteorological factors and mumps in Jining, China. *The Science of the total environment* **568** (2016).

13 Runzi, L. *et al.* Association between incidence of mumps and meteorological factors in Ji'ning. *Journal of Environment and Health* **33** (2016).

14 Li, W. Discussion on the relationship between weather conditions and mumps in Zhangjiajie city. *Statistics and Management*, 52-53 (2016).

15 Jing, L., Zhi, Z., Shaili, J. & Guangjun, W. Analysis of relationship between mumps and meteorological conditions in Yinchuan city. *Journal of Ningxia Medical University* **37** (2015).

16 Huanmiao, X. *Impacts of Tropical Cyclones on Infectious Diseases in Guangdong Province, 2005-2011*, Shandong University, (2015).

17 Zhi, Z., Jing, L. & Shaoping, J. The Epidemic Characteristics of Mumps and Relations with Meteorological Factors in Yinchuan. *Journal of Arid Meteorology* **33**, 162-166 (2015).

18 Yang, Q. *et al.* The relationship between meteorological factors and mumps incidence in Guangzhou, China, 2005–2012. *Human vaccines & immunotherapeutics* **10** (2014).

19 Chuanxi, F. *et al.* Time series study of relationship between meteorological factors and mumps incidence. *South China J Prev Med* **40** (2014).

20 Xiaojuan, W. & Xiaoli, Z. Correlation study and prediction model analysis of key infectious diseases and climate in Linyi City. *Journal of Community Medicine*, 7 (2014).

21 Xuan, Z. & Juan, H. Correlation between meteorological factors and mumps incidence in Beijing from 1990 to 2004. *Chinese Journal of Traditional Chinese Medicine and Pharmacy* **29** (2014).

22 Baihua, S. *Correlation between mumps infection and climatic factors in Taiwan* Master thesis, Cheng Kung University, (2013).

23 Shaoping, J., Xu, L., Zhi, Z. & Jing, L. Relationship between mumps and climatic factors in Yinchuan city. *Journal of Environment and Health* **30** (2013).

24 Chao, Y. *et al.* Study on Cold Area of Meteorological Factors to the Impact Ofurban Main Infectious Diseases Paleolimnologic. *Chinese Primary Health Care* **25**, 79-82 (2011).
